# Regulatorische Einordnung KI-basierter Produkte für die medizinische Anwendung auf Basis von EU AI Act und MDR/IVDR

**DOI:** 10.1007/s00103-025-04091-9

**Published:** 2025-07-04

**Authors:** Steffen Luckner, Wolfgang Lauer

**Affiliations:** 1 Referat 124 – Medizinproduktesicherheit, Bundesministerium für Gesundheit, Mauerstraße 29, 10117 Berlin, Deutschland; 2 Bundesinstitut für Arzneimittel und Medizinprodukte (BfArM), Bonn, Deutschland

**Keywords:** Künstliche Intelligenz, Medizinprodukte, MDR, AI Act, Risikoklassifizierung, Artificial intelligence, Medical devices, MDR, AI Act, Risk classification

## Abstract

Der Einsatz künstlicher Intelligenz (KI) im Gesundheitswesen bietet großes Potenzial, bringt jedoch auch regulatorische Herausforderungen mit sich. Der „Artificial Intelligence Act“ der Europäischen Union („EU AI-Act“, kurz: AIA), die „Medical Device Regulation“ (MDR) und die „Regulation on in vitro diagnostic medical devices“ (IVDR) legen den Rahmen für die sichere und ethische Nutzung KI-basierter Medizinprodukte in Europa fest.

Bei der regulatorischen Einordnung von KI-Software oder Produkten mit KI-Elementen ist anhand der vom Hersteller festgelegten Zweckbestimmung und der Abgrenzungskriterien der MDR bzw. IVDR zu klären, ob es sich um ein Medizinprodukt handelt. Dann gelten alle entsprechenden Regelungen des AIA zusätzlich zu denen der MDR/IVDR.

Sowohl in der MDR/IVDR als auch im AIA werden Produkte nach ihrem Risikopotenzial Risikoklassen zugeordnet und auf dieser Basis für sie geltende Anforderungen definiert. Die Kriterien und Konsequenzen unterscheiden sich jedoch zwischen den beiden Regelungsbereichen, sodass z. B. auch nach MDR/IVDR niedrigklassifizierte Medizinprodukte mit Beteiligung einer Benannten Stelle gemäß AIA als „Hochrisikoprodukte“ gelten. Daher ist es sehr wichtig, genau zwischen den jeweiligen Regelungen zu unterscheiden.

Die Harmonisierung regulatorischer Vorgaben bleibt eine Herausforderung, insbesondere da die Klassifizierung nach AIA und MDR/IVDR trotz teilweise ähnlicher Begriffe auf unterschiedlichen Kriterien basiert. Offene Fragen bestehen v. a. bei Medizinprodukten der niedrigsten Risikoklasse und selbstlernenden KI-Systemen. Die Weiterentwicklung regulatorischer Leitlinien ist notwendig, um innovative KI-Anwendungen sicher und effizient in die Gesundheitsversorgung zu integrieren. Das Bundesministerium für Gesundheit (BMG) und das Bundesinstitut für Arzneimittel und Medizinprodukte (BfArM) engagieren sich dazu auf nationaler und europäischer Ebene für pragmatische Lösungen.

## Abstract (en)

The use of artificial intelligence (AI) in healthcare offers great potential but also presents regulatory challenges. The EU* Artificial Intelligence Act* (AIA), the *Medical Device Regulation* (MDR), and the *Regulation on in vitro diagnostic medical devices* (IVDR) establish the framework for the safe and ethical use of AI-based medical devices in Europe.

When assessing the regulatory classification of AI software or products with AI components, it must be determined—based on the intended purpose defined by the manufacturer and the classification criteria of the MDR or IVDR—whether the product qualifies as a medical device. If so, all relevant regulations of the AIA apply in addition to those of the MDR/IVDR.

Both the MDR/IVDR and the AIA assign products to different risk classes based on their respective risk potential, defining specific requirements accordingly. However, the classification criteria and implications differ between the regulatory frameworks. As a result, even medical devices in the lower MDR/IVDR risk category with involvement of a notified body are considered high-risk products under the AIA. It is therefore crucial to differentiate precisely between the respective regulations.

The harmonization of regulatory requirements remains a challenge, particularly because classification under the AIA and MDR/IVDR is based on different criteria despite some similar terminology. Open questions persist, especially regarding lowest-risk medical devices and self-learning AI systems. Further development of regulatory guidelines is necessary to ensure the safe and efficient integration of innovative AI applications into healthcare. The German Federal Ministry of Health (BMG) and the Federal Institute for Drugs and Medical Devices (BfArM) are actively working at both national and European levels to develop pragmatic solutions.

## Einleitung

Die Anwendung von künstlicher Intelligenz (KI) hat auch im Gesundheitswesen in den vergangenen Jahren für großes Interesse gesorgt. Zahlreiche Untersuchungen wurden in den letzten Jahren über den allgemeinen Einsatz von KI in der Versorgung von Patientinnen und Patienten sowie zu ganz konkreten Einsatzzwecken veröffentlicht [1–3]. Insgesamt wird der KI-Technologie eine hohe Bedeutung und enormes Potenzial für die gesamte Gesundheitsversorgung zugesprochen [1, 2, 4].

Dabei ist KI kein Thema nur der letzten Jahre. Sucht man zum Beispiel in der englischsprachigen Metadatenbank PubMed nach dem Stichwort „Artificial Intelligence“ werden über 115.000 Ergebnisse angezeigt. Die ersten Artikel im Zusammenhang mit KI reichen bis in das Jahr 1961 zurück.^1^ Seit den 2000er-Jahren haben die Publikationen zum Thema KI zahlenmäßig zugenommen, mit einer deutlichen, kontinuierlichen Zunahme seit dem Ende der 2010er-Jahre. Dies spiegelt sowohl den enormen technologischen Fortschritt in diesem Bereich als auch das hohe Interesse an der Thematik und ihrer praktischen Anwendung, auch in der Gesundheitsversorgung, wider.

Der zunehmende Einsatz von KI im Gesundheitswesen wurde durch eine Kombination aus technologischen, regulatorischen und gesellschaftlichen Entwicklungen begünstigt. Die stetige Verbesserung von Datenverarbeitungskapazitäten, die Entwicklung leistungsstarker Algorithmen und der Zugang zu großen, auswertbaren Datenmengen bilden eine wesentliche Grundlage für die Entwicklung moderner KI-Anwendungen [5–7].

Vor allem Fortschritte in der Bild- und Textverarbeitung haben die Anwendung von KI im Gesundheitswesen erheblich vorangetrieben, beispielsweise im Bereich der medizinischen Bildgebung [7]. Dabei wird der KI das Potenzial zugesprochen, das Gesundheitswesen tiefgreifend zu verändern. Von der Diagnose seltener Erkrankungen über die personalisierte Medizin bis hin zur Optimierung von Krankenhausabläufen – KI-basierte Anwendungen eröffnen vielfältige Möglichkeiten, die Qualität, Patientensicherheit und Effizienz der medizinischen Versorgung zu verbessern [2, 7].

KI-Systeme als Medizinprodukt sind seit einigen Jahren zunehmend auf dem Markt erhältlich. Beispielsweise führt die US-amerikanische Behörde für Lebens- und Arzneimittel (FDA) in einer Liste von in den USA zugelassenen Medizinprodukten mit KI oder maschinellem Lernen (ML) über 1000 Produkte [8]. Bei diesen handelt es sich überwiegend um Expertensysteme oder Systeme mit effizienteren Berechnungsmethoden, die vor allem im Bereich der Diagnostik und Befundung eingesetzt werden. Weitere Anwendungsfälle finden sich z. B. in den Bereichen prädiktive Risikoanalyse, personalisierte Medizin und klinische Entscheidungsunterstützungssysteme sowie Vorhersagemodelle. Für diese Anwendungsfälle haben sich kommerzielle KI-Anwendungen allerdings noch nicht vollständig etabliert.

Der Einsatz von KI bietet große Chancen, kann jedoch auch zu Herausforderungen führen und neue Risiken z. B. im Hinblick auf die Sicherheit und die Einhaltung der Grundrechte mit sich bringen. Vor diesem Hintergrund hat der Europäische Gesetzgeber mit der Verordnung (EU) 2024/1689 über künstliche Intelligenz, den EU AI Act (im Text kurz: AIA; [9]), einen horizontalen Rechtsrahmen beschlossen, der die Voraussetzung für die Entwicklung, Verwendung und Überwachung vertrauenswürdiger KI in der Union schafft.

Auch die Frage, ob bzw. unter welchen Voraussetzungen z. B. große Sprachmodelle (Large Language Models, LLM), wie ChatGPT oder DeepSearch, die grundsätzlich auch Fragen zu medizinischen Inhalten beantworten können, als Medizinprodukt einzuordnen sind und somit unter die Regelungen der Verordnung (EU) 2017/745 („Medical Device Regulation“, MDR; [10]) bzw. der Verordnung (EU) 2017/746 („Regulation on in vitro diagnostic medical devices“, IVDR; [11]) und die entsprechenden nationalen Gesetze und Verordnungen fallen, stellt sich mit diesen Entwicklungen.

Im vorliegenden Beitrag wird die regulatorische Einordnung von Produkten mit KI-Elementen für die medizinische Anwendung auf Basis des AIA und der MDR bzw. IVDR beschrieben.

## Der AIA zur Regulierung von Produkten mit künstlicher Intelligenz

Der Grundstein für den AIA wurde 2017 gelegt, als der Europäische Rat ein Bewusstsein für die Auseinandersetzung mit neuen Trends wie künstlicher Intelligenz für den Aufbau eines digitalen Europas forderte, um neue Märkte zu erschließen und die Führungsrolle der europäischen Industrie in diesen Bereichen zu bestätigen [12]. Der von der Europäischen Kommission 2018 vorgelegte Plan für künstliche Intelligenz („AI made in Europe“) wurde vom Europäischen Rat 2019 angenommen und rief die Europäische Kommission auf, bestehende Rechtsvorschriften der EU im Hinblick auf die Entwicklung sicherer, vertrauenswürdiger und ethisch vertretbarer KI zu überprüfen [13].

Die europäische KI-Strategie von 2018 zielte darauf ab, Forschungs- und Industriekapazitäten zu stärken, die Vorteile der KI-Technologie zu nutzen und gleichzeitig Sicherheit und Grundrechte zu gewährleisten [13]. In 2020 wurden zudem vom Europäischen Rat eine einheitliche Definition für Anwendungen mit künstlicher Intelligenz und die Adressierung von Risiken im Zusammenhang mit der Technologie gefordert. Die Ratsschlussfolgerungen enthalten z. B. die Forderung, dass entsprechende Anwendungen mit den Grundrechten der EU vereinbar sind, und thematisierten u. a. Verzerrung (Bias), Unberechenbarkeit und autonomes Verhalten [14, 15]. Um diese Ziele zu erreichen, hat die Europäische Kommission 2021 ein KI-Paket vorgelegt, das auch den Entwurf des AIA umfasste [16]. Der AIA ist dabei auch eine Reaktion auf die wiederholten Forderungen des Europäischen Parlaments und des Europäischen Rates an die Europäische Kommission, legislative Maßnahmen für einen funktionierenden EU-Binnenmarkt für Systeme mit KI unter gleichzeitiger Berücksichtigung des Nutzens und der Risiken der Technologie zu unternehmen [17]. Da bereits in der KI-Strategie die Förderung von KI-Anwendungen in Schlüsselbereichen wie dem Gesundheitswesen gefordert wurde, ist es nicht verwunderlich, dass der AIA auch Medizinprodukte und weitere Anwendungsszenarien im Gesundheitswesen mitberücksichtigt.

Der AIA regelt die Entwicklung und Nutzung von KI horizontal über mehrere bestehende Produktvorschriften, wie z. B. Maschinen‑, Finanz- und Medizinprodukte, sowie für neue Bereiche, wie zum Beispiel für die biometrische Identifizierung und Kategorisierung von Personen, im Bildungsbereich und in der Strafverfolgung. Dafür enthält der Entwurf unter anderem Definitionen, Klassifizierungsregeln, grundlegende Anforderungen an sogenannte Hochrisiko-KI, Pflichten für Wirtschaftsakteure sowie Regelungen zur Benennung von und Anforderungen an Benannte Stellen, zum Konformitätsbewertungsverfahren sowie zur Marktüberwachung. Ein weiteres wichtiges Element sind innovationsfördernde Maßnahmen u. a. zum Testen und Trainieren von KI-Anwendungen unter kontrollierten Umgebungen (sogenannte KI-Reallabore). Der AIA folgt grundsätzlich einem risikobasierten Regulierungsansatz. Den nachfolgenden Risikoklassen sollen KI-Systeme zugeordnet werden:

- *unannehmbares Risiko:* KI-Systeme, die als klare Bedrohung für die Sicherheit, die Lebensgrundlagen und die Rechte der Menschen gelten, werden verboten (z. B. Social Scoring für Anspruch auf wesentliche Gesundheitsdienstleistung, die eine ungerechtfertigte Schlechterstellung oder Benachteiligung haben);
- *hohes Risiko:* KI-Systeme, bei denen ein hohes Risiko besteht, wenn KI-Technik in bestimmten Bereichen eingesetzt wird; Produktvorschriften, die in Annex I (u. a. MDR und IVDR), und Bereiche, die in Annex III (z. B. für die Entsendung von Not- und Rettungsdiensten) aufgelistet sind, gelten als Hochrisikoprodukte;
- *geringes Risiko:* KI-Systeme, für die besondere Transparenzverpflichtungen gelten (z. B. Chatbot zur Unterstützung beim Ausfüllen eines Pflegeantrags);
- *kein oder minimales Risiko:* keine zusätzliche Regulierung notwendig (z. B. Spamfilter).

Medizinprodukte und In-vitro-Diagnostika (IVD) gelten nach dem AIA in den allermeisten Fällen als Hochrisiko-KI [9, 18–20].

## Abgrenzung von Produkten mit KI-Elementen als Medizinprodukt

Um die Frage zu beantworten, ob eine Anwendung mit KI-Elementen als Medizinprodukt anzusehen ist, muss überprüft werden, ob das konkrete Produkt mit der durch seinen Hersteller festgelegten Zweckbestimmung die Definition eines Medizinproduktes erfüllt. Dabei handelt es sich nach den in der EU geltenden rechtlichen Vorgaben immer um eine Einzelfallentscheidung bezogen auf ein konkretes Produkt (z. B. eine Software) in einer bestimmten Anwendung.

Die gesetzliche Grundlage für eine entsprechende Abgrenzungsentscheidung bilden die in Artikel 2 Nummer 1 und Nummer 12 der MDR genannten Begriffsbestimmungen für „Medizinprodukt“ und „Zweckbestimmung“ (siehe Infobox 1; [10]):

Eine zentrale Voraussetzung dafür, dass es sich bei einer Software um ein Medizinprodukt handelt, ist, dass ihr bei Verwendung entsprechend den Angaben des Herstellers eine medizinische Zweckbestimmung nach der Begriffsdefinition eines Medizinproduktes in der MDR zukommt. Im Leitfaden „MDCG 2019-11“ der „Medical Device Coordination Group“ (MDCG) wird dabei von einer „Medical Device Software“ (MDSW) gesprochen [21].

Der Leitfaden MDCG 2019-11 gibt an, dass eine Software neben einer medizinischen Zweckbestimmung weitere Bedingungen erfüllen muss, um den Regelungen für Medizinprodukte zu unterfallen.

Weitere Fragestellungen sind z. B.:

- Liegt eine Zweckbestimmung vor, die über die reine Speicherung, Archivierung oder verlustfreie Komprimierung, Kommunikation oder eine einfache Suche von Daten hinausgeht?
- Bezieht sich der vorgesehene Einsatz auf den Nutzen für einen individuellen Patienten?

Als Beispiele für Software, die nicht zum Nutzen eines individuellen Patienten angesehen wird, nennt der Leitfaden MDCG 2019-11 u. a. Software, die nur dazu bestimmt ist, Populationsdaten zu aggregieren, Diagnose- und Behandlungspfade aufzuzeigen, die nicht auf einen individuellen Patienten ausgerichtet sind, oder auch Software, die nur für epidemiologische Studien oder Register bestimmt ist.

Demgegenüber unterliegt der Regulation als Medizinprodukt jedoch Software,

- die dazu bestimmt ist, ein Medizinprodukt zu steuern oder dessen Anwendung zu beeinflussen,
- die vom Hersteller als „Zubehör“ zu einem Medizinprodukt nach Artikel 2 Nummer 2 MDR bzw. IVD nach Artikel 2 Nummer 4 IVDR bestimmt ist oder
- die zu der Gruppe von Produkten ohne medizinische Zweckbestimmung nach Anhang XVI MDR gehört.

Dabei ist nicht maßgeblich, ob die Software selbst eine medizinische Zweckbestimmung hat.

Für die Einordnung einer Software als IVD, einer Untergruppe von Medizinprodukten, muss sie zusätzlich die Definition für ein IVD entsprechend der Begriffsdefinition nach Artikel 2 Nummer 2 IVDR erfüllen [11].

Um die Anwendung der Regelungen in der Praxis zu unterstützen, bietet der Leitfaden MDCG 2019-11 vielfältige Informationen und Erläuterungen, u. a. einen Entscheidungsbaum zur Abgrenzung von „Medical Device Software“ und viele Beispiele.

Darüber hinaus bietet auch das kontinuierlich erweiterte „Manual on borderline and classification in the Community Regulatory framework for medical devices“ eine Orientierungshilfe durch konkrete Abgrenzungsbeispiele [22]. Weitere Unterstützung bieten z. B. Entscheidungshilfen europäischer Medizinproduktebehörden, wie z. B. die Orientierungshilfe des BfArM [23].

Auch bei Produkten mit KI-Elementen oder bei KI-Software selbst ist eine Abgrenzungsentscheidung also immer für das individuelle Produkt auf Basis der von seinem Hersteller festgelegten Zweckbestimmung im Abgleich mit der Begriffsdefinition in der MDR bzw. IVDR zu treffen. Dabei ist sowohl die konkret formulierte Zweckbestimmung zu berücksichtigen als auch u. a. die Gebrauchsinformationen und Werbematerialien zum Produkt, im Falle von KI ggf. auch entsprechende Hinweise und Einordnungen der Antworten durch die Anwendung selbst, im Sinne der Konsistenz zwischen Zweckbestimmung und Produktverhalten.

Allein die Tatsache, dass es aufgrund der Produktbeschaffenheit grundsätzlich möglich ist, ein Produkt auch für einen medizinischen Zweck einzusetzen, macht das Produkt nicht zu einem Medizinprodukt. Dies lässt sich am Beispiel eines Schweizer Messers veranschaulichen, das mit seinen vielfältigen Werkzeugen technisch auch für medizinische Anwendungen genutzt werden könnte, dafür aber in aller Regel nicht zweckbestimmt und insofern nicht als Medizinprodukt anzusehen ist.

## Risikoklassifizierung nach MDR/IVDR und EU AI Act

Um die große Bandbreite von Produkten für die Anwendung gesetzlicher Regelungen zu strukturieren, hat der Gesetzgeber z. B. in verschiedenen Zusammenhängen Risikoklassen definiert. Darin werden jeweils Produkte mit einem nach bestimmten Kriterien vergleichbaren Risikopotenzial zusammengefasst, um jeweils angemessene Anforderungen z. B. für das Inverkehrbringen zuzuordnen.

Sowohl in der MDR bzw. IVDR als auch im AIA werden entsprechend Risikoklassen definiert, allerdings mit unterschiedlichen Kriterien. Daher muss klar zwischen den jeweiligen Klassifizierungen differenziert werden.

### Risikoklassifizierung nach MDR/IVDR

Nach Artikel 51 MDR werden Medizinprodukte unter Berücksichtigung der vom Hersteller festgelegten Zweckbestimmung und der damit verbundenen Risiken in die Klassen I (s, m, r), IIa, IIb und III eingestuft, wobei Klasse I das geringste Risikopotenzial abbildet, Klasse III das höchste. Die Klassifizierung erfolgt gemäß den Klassifizierungsregeln im Anhang VIII der MDR und adressiert insbesondere das Risikopotenzial durch direkte oder indirekte Auswirkungen auf die Gesundheit von Patienten, Anwendern oder Dritten (z. B. durch ein gebrochenes künstliches Hüftgelenk, eine Infektion einer künstlichen Herzklappe oder eine unangemessene therapeutische Entscheidung auf Basis einer falschen Information aus einer diagnostischen Software).

Software gilt nach den Begriffsbestimmungen in Artikel 2 Nummer 4 MDR als „aktives Medizinprodukt“. Neben den Klassifizierungsregeln 9, 10, 11, 12 und 13 für aktive Medizinprodukte sind auch weitere Klassifizierungsregeln von Relevanz für Software, beispielsweise Regel 15 für Produkte, die zur Empfängnisverhütung oder zum Schutz vor der Übertragung von sexuell übertragbaren Krankheiten eingesetzt werden, und Regel 22 für Produkte z. B. mit geschlossenen Regelsystemen.

Im Gegensatz zu den Regeln 9 und 10, die bei allen aktiven therapeutischen bzw. aktiven diagnostischen Medizinprodukten Anwendung finden, adressiert Regel 11 speziell die Klassifizierung von Software ([10]; siehe Infobox 2):

Im Leitfaden MDCG 2019-11 wird empfohlen, die Risikoeinstufung für den ersten Absatz der Regel 11 in Analogie zum „Risk Framework“ des „International Medical Device Regulators Forum“ (IMDRF; [24]) zu sehen. Darüber hinaus listet das fortlaufend erweiterte „Manual on borderline and classification in the Community Regulatory framework for medical devices“ konkrete Klassifizierungsbeispiele [22].

Die Klassifizierung von Software als IVD in die Klassen A, B, C und D erfolgt gemäß Artikel 47 Nummer 1 IVDR unter Berücksichtigung der vom Hersteller vorgegebenen Zweckbestimmung und der damit verbundenen Risiken nach den Klassifizierungsregeln im Anhang VIII IVDR.

### Risikoklassifizierung nach AIA

Die Einstufung eines KI-Medizinproduktes nach AIA als „hochriskant“ bedeutet nicht zwangsläufig, dass von diesem Produkt auch ein hohes Risiko nach MDR/IVDR ausgeht. Dieses Missverständnis kommt unter anderem durch den verwendeten Begriff „Hochrisiko-KI-System“ zustande. Vor dem Hintergrund, dass auch nach MDR/IVDR niedrig klassifizierte Produkte (Klasse Is, Im, Ir und As) als Hochrisiko-KI-System gelten können, ist die Wirkungsweise des „risikobasierten“ Ansatzes des AIA grundsätzlich zu hinterfragen. Vielmehr handelt es sich bei Hochrisiko-KI-Systemen um Produkte, die nach Willen des europäischen Gesetzgebers aufgrund ihrer Anwendung besonders prüfbedürftig sind bzw. für die zusätzliche Anforderungen gelten.

Für die Klassifizierung als Hochrisiko-KI-System nach AIA ist zunächst zu überprüfen, ob das Produkt unter die Definition eines KI-Systems und damit unter den AIA fällt. Hierzu sollte die Hilfestellung der Europäischen Kommission herangezogen werden,^2^ denn nicht jedes KI-fähige Produkt ist ein KI-System im Sinne des AIA. Gleichwohl nach Absatz 39 dieser Hilfestellung zahlreiche KI-fähige Produkte als KI-System zu betrachten sind, vor allem medizinische Expertensysteme der ersten Generation, die auf logik- und wissensbasierten Ansätzen beruhen.

Artikel 6 AIA enthält die für KI-fähige Produkte relevante Regelung für die Klassifizierung. Im Nachfolgenden werden nur die für Medizinprodukte relevanten Regelungen des Artikels 6 Absatz 1 AIA näher beleuchtet. Die erste Bedingung nach Artikel 6 Absatz 1 a) AIA ist leider missverständlich formuliert. Die Erwägungsgründe 50, 51 und 52 stellen klar, dass hier KI-Systeme gemeint sind, die Sicherheitsbauteile von Produkten sind oder selbst ein Produkt sind und einem Rechtsakt unter Anhang I AIA fallen. Der Begriff des Sicherheitsbauteils kommt aus der europäischen Maschinen-Verordnung, die im Juni 2023 vom Rat der Europäischen Union und dem Europäischen Parlament verabschiedet wurde. Bei Medizinprodukten ist derzeit nicht ganz klar, was unter einem Sicherheitsbauteil konkret zu verstehen ist. Eine Überschneidung mit einer Medizinproduktesoftware, die ein Medizinprodukt steuert oder dessen Gebrauch beeinflusst, erscheint am wahrscheinlichsten. Das KI-System unterliegt der MDR oder IVDR, wenn es sich dabei um eine Medizinproduktesoftware, ein Zubehör zu einem Medizinprodukt, ein Annex-XVI-Produkt^3^ handelt oder ein (Hardware‑)Medizinprodukt steuert oder beeinflusst (siehe vorheriger Abschnitt zur Risikoeinstufung nach MDR/IVDR).

Auch die zweite Bedingung nach Artikel 6 Absatz 1 b) AIA ist, mindestens in der deutschen Übersetzung, missverständlich formuliert. Auch hier hilft ein Blick in die Erwägungsgründe 50 bis 52. KI-Systeme, an deren Konformitätsbewertungsverfahren eine Benannte Stelle beteiligt werden muss, gelten nach Artikel 6 Absatz 1 b) AIA als hochriskant. Da bei Produkten, die gemäß Artikel 5 Absatz 5 MDR/IVDR von Gesundheitseinrichtungen hergestellt und verwendet werden (Eigenherstellung), keine Benannte Stelle zu beteiligen ist, gelten eigenhergestellte KI-Systeme nicht als Hochrisiko-KI-System. Für die Klassifizierung von KI-Medizinprodukten muss betrachtet werden, ob eine Benannte Stelle zu beteiligen ist. Nach Artikel 52 MDR ist bei Medizinprodukten der Risikoklassen Is, Im, Ir, IIa, IIb und III sowie nach Artikel 48 IVDR bei IVD der Risikoklassen As, B, C und D eine Benannte Stelle zu beteiligen. Vor dem Hintergrund der Regel 11 Anhang VIII MDR ist davon auszugehen, dass der überwiegende Teil der KI-Medizinprodukte mindestens als Klasse IIa zu klassifizieren ist und damit als Hochrisiko-KI-System gelten wird. Fraglich ist auch, ob es eine signifikante Anzahl an KI-Medizinprodukten der Klasse Ir, Is, Im und As am Markt geben wird. Hersteller müssen entscheiden, ob der Aufwand für die Entwicklung sowie das Trainieren, Validieren und Testen des KI-Algorithmus für Klasse-I-KI-Medizinprodukte überhaupt angemessen ist oder sich die beabsichtigte Zweckbestimmung nicht besser durch eine Software erreichen lassen kann, die keine KI enthält.

**Abb. 1:**
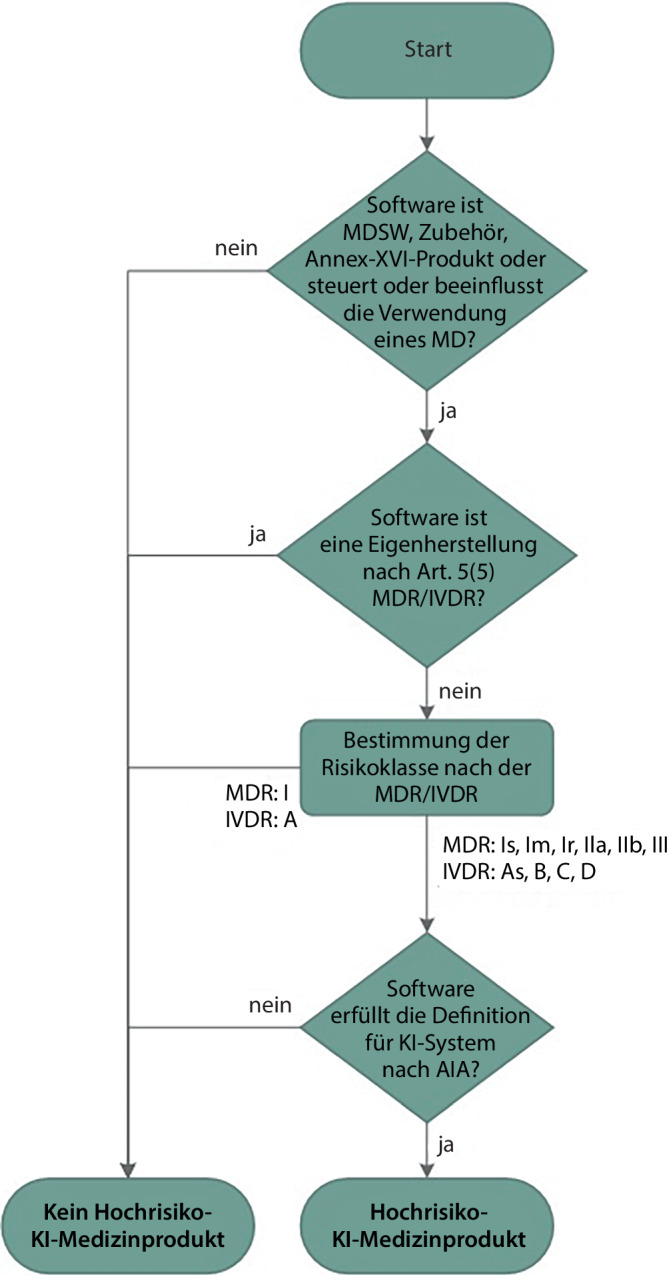
Entscheidungsbaum für die Klassifizierung von Medizinprodukten als Hochrisiko-KI-System nach dem EU AI Act (AIA). (*MDSW* Medical Device Software, *MD* Medical Device, *MDR* Medical Device Regulation, *IVDR* In Vitro Diagnostic Regulation, *AIA* EU AI Act)

In der Abb. 1 ist der Prüfprozess für die Klassifizierung von Hochrisiko-KI-Medizinprodukten nach AIA zusammengefasst.

## Datenschutz als Teil der grundlegenden Anforderungen

Für die Entwicklung von KI-Systemen sind qualitativ hochwertige Daten und der Zugang zu diesen ein zentrales Element, v. a. für KI-Systeme, die mit Daten trainiert werden. In Artikel 10 AIA sind umfangreiche Anforderungen an die Daten und das Datenmanagement beschrieben, die sicherstellen sollen, dass die notwendigen Daten für das Trainieren, Valideren und Testen gesammelt, gelabelt, bewertet, dokumentiert und versioniert werden.

Dem Schutz personenbezogener Daten kommt eine besondere Rolle zu, insbesondere weil KI-Systeme, vor allem im Gesundheitswesen, sensible Daten verarbeiten, die direkten Einfluss auf die Grundrechte der betroffenen Personen haben. Der AIA betont an mehreren Stellen, dass Datenschutz nicht nur ein ergänzendes Kriterium ist, sondern auch Teil der grundlegenden Anforderungen von Hochrisiko-KI-Systemen.

Vor dem Hintergrund von Risiken, die mit unzureichenden Datenschutzmaßnahmen in Hochrisiko-KI-Systemen einhergehen können, hebt der AIA die Notwendigkeit hervor, Datenschutz von Anfang an als integralen Bestandteil des gesamten Lebenszyklus des KI-Systems in den Entwicklungsprozess einzubinden. Das bedeutet auch, dass bereits bei der Konzeption und während des Trainings, Validierens und Testens von Modellen strenge Datenschutzprinzipien berücksichtigt werden müssen. Datenschutz ist daher nicht isoliert zu betrachten, sondern als Bestandteil des Risikomanagements, etwa durch Konzepte wie „privacy by design“ und „privacy by default“. Alle datenverarbeitenden Prozesse sollten transparent gestaltet und dokumentiert werden. Dadurch wird es möglich, den Umgang mit personenbezogenen Daten jederzeit nachzuvollziehen und bei Bedarf gezielte Maßnahmen zu ergreifen. Es wird implizit darauf verwiesen, dass die Anforderungen des AIA grundsätzlich in Einklang mit den Vorgaben der Datenschutzgrundverordnung (DSGVO) stehen müssen. Artikel 10 Absatz 5 AIA enthält dabei eine Ausnahme für die Verarbeitung besonderer Kategorien von personenbezogenen Daten, wenn diese für die Erkennung und Korrektur von (unerwünschten) Verzerrungen (Bias) erforderlich ist.

Neben den technischen Voraussetzungen sind auch Betreiber von Hochrisiko-KI-Systemen verpflichtet, Anforderungen im Hinblick auf den Datenschutz umzusetzen und Datenschutzaspekte in den Betrieb zu integrieren (siehe Artikel 29 AIA). Diese Maßnahmen sollen sicherstellen, dass personenbezogene Daten auf Betreiberseite geschützt werden und gleichzeitig die Transparenz und Nachvollziehbarkeit der KI-Systeme gewährleistet wird.

## Diskussion und Fazit

Im Zuge der enormen technologischen Entwicklung von Ansätzen künstlicher Intelligenz (KI) halten entsprechende Produkte zunehmend auch in die Gesundheitsversorgung Einzug und bieten großes Potenzial, z. B. beim Wissensmanagement und der Entscheidungsunterstützung. KI-Software kann je nach Zweckbestimmung im Abgleich mit der Medizinproduktedefinition gemäß der Verordnung (EU) 2017/745 („Medical Device Regulation“, MDR) selbst ein Medizinprodukt oder Bestandteil von Medizinprodukten sein.

Die Risikoklassifizierung von Medizinprodukten mit KI-Elementen ist nicht trivial und ebenfalls maßgeblich von der individuellen Zweckbestimmung abhängig. Darüber hinaus ist das Zusammenwirken der Verordnungen (EU) 2024/1689 über künstliche Intelligenz (den EU AI Act, kurz: AIA), MDR und Verordnung (EU) 2017/746 („Regulation on in vitro diagnostic medical devices“, IVDR) zu berücksichtigen sowie die Tatsache, dass die Zuordnung von Medizinprodukten zu Risikoklassen in der MDR und IVDR einerseits und dem AIA andererseits nach unterschiedlichen Kriterien erfolgen und unterschiedliche Konsequenzen haben kann. Eine differenziertere Klassifizierung nach AIA wäre wünschenswert gewesen. Aufgrund des Zusammenspiels mit den Klassifizierungsregeln nach MDR/IVDR wird ein überwiegender Teil kommerzieller KI-Medizinprodukte als Hochrisiko-KI-System gelten, v. a. bei niedrig klassifizierten KI-Medizinprodukten.

Die Anforderungen im AIA zum Datenschutz stellen sicher, dass die Datengrundlage eines Hochrisiko-KI-Systems hohen Qualitätsanforderungen genügt, kontinuierlich überwacht wird und bei Bedarf Maßnahmen ergriffen werden können. Um die technologischen Anforderungen erfüllen zu können, bietet der AIA die notwendigen Ausnahmen (siehe Artikel 10 Absatz 5 AIA).

Am Beispiel der Klassifizierung ist stellvertretend erkennbar, dass das Zusammenspiel des AIA mit der MDR und IVDR regulatorisch nicht ganz klar ist. Umfangreiche Erläuterungen werden voraussichtlich nötig sein, um die zahlreichen ausstehenden Fragen beantworten zu können, insbesondere die Einhaltung von Anforderungen aus unterschiedlichen EU Rechtsvorschriften (vgl. Artikel 8 Absatz 2 AIA).

Zahlreiche Fragen insbesondere im Hinblick auf Klasse-I-Medizinprodukte und Klasse-A-IVD, etwa hinsichtlich eines abweichenden Geltungsbeginns oder des Umgangs mit weiteren Anforderungen aus dem AIA, sind derzeit noch völlig offen. Zudem müssen noch fehlende Konzepte für selbstlernende KI-Medizinprodukte oder die klinische Bewertung von KI-Medizinprodukten erarbeitet werden. Hersteller von KI-Medizinprodukten stehen großen Implementierungsherausforderungen gegenüber, insbesondere kleine und mittlere Unternehmen (KMU) und Start-ups. Vor dem Hintergrund der Implementierung der MDR und IVDR, die parallel noch andauert, werden Unternehmen Ressourcen und Kapazitäten ggf. eher in den Bereich Regulatory Affairs und die Erfüllung der gesetzlichen Anforderungen investieren statt in Innovationen.

Bei einer strikteren Anwendung der interinstitutionellen Vereinbarung zwischen Europäischer Kommission, Europäischem Parlament und Europäischem Rat über bessere Rechtsetzung hätten Unklarheiten vermieden werden können [25].

Das Bundesministerium für Gesundheit (BMG) und das Bundesinstitut für Arzneimittel und Medizinprodukte (BfArM) arbeiten gemeinsam mit den anderen beteiligten Institutionen und den Industrieverbänden in Deutschland sowie der EU-Kommission und den Gremien auf europäischer Ebene intensiv an einer effektiven und effizienten Umsetzung des AIA für Medizinprodukte, z. B. bei der Erstellung von EU-Leitlinien, sowie an einer innovationsfreundlichen und bürokratiearmen nationalen Umsetzung des AIA. Um entsprechende Zukunftstechnologien möglichst schnell und pragmatisch in der Gesundheitsversorgung in Deutschland zu etablieren, wird der Dialog fortgeführt und entsprechende Informationen auf den Webseiten veröffentlicht.

### Infobox 1 Begriffsbestimmungen für „Medizinprodukt“ und „Zweckbestimmung“ gemäß Verordnung (EU) 2017/745 über Medizinprodukte (Medical Device Regulation, MDR)

Artikel 2 Nummer 1 MDR:

„Medizinprodukt“ bezeichnet ein Instrument, einen Apparat, ein Gerät, eine Software, ein Implantat, ein Reagenz, ein Material oder einen anderen Gegenstand, das dem Hersteller zufolge für Menschen bestimmt ist und allein oder in Kombination einen oder mehrere der folgenden spezifischen medizinischen Zwecke erfüllen soll:

- Diagnose, Verhütung, Überwachung, Vorhersage, Prognose, Behandlung oder Linderung von Krankheiten,
- Diagnose, Überwachung, Behandlung, Linderung von oder Kompensierung von Verletzungen oder Behinderungen,
- Untersuchung, Ersatz oder Veränderung der Anatomie oder eines physiologischen oder pathologischen Vorgangs oder Zustands,
- Gewinnung von Informationen durch die In-vitro-Untersuchung von aus dem menschlichen Körper – auch aus Organ‑, Blut- und Gewebespenden – stammenden Proben

und dessen bestimmungsgemäße Hauptwirkung im oder am menschlichen Körper weder durch pharmakologische oder immunologische Mittel noch metabolisch erreicht wird, dessen Wirkungsweise aber durch solche Mittel unterstützt werden kann.

Die folgenden Produkte gelten ebenfalls als Medizinprodukte:

- Produkte zur Empfängnisverhütung oder -förderung,
- Produkte, die speziell für die Reinigung, Desinfektion oder Sterilisation der in Artikel 1 Absatz 4 genannten Produkte und der in Absatz 1 dieses Spiegelstrichs genannten Produkte bestimmt sind.

Artikel 2 Nummer 12 MDR:

„Zweckbestimmung“ bezeichnet die Verwendung, für die ein Produkt entsprechend den Angaben des Herstellers auf der Kennzeichnung, in der Gebrauchsanweisung oder dem Werbe- oder Verkaufsmaterial bzw. den Werbe- oder Verkaufsangaben und seinen Angaben bei der klinischen Bewertung bestimmt ist.

### Infobox 2 Regel 11 über die Risikoklassifizierung von Software gemäß Annex VIII Verordnung (EU) 2017/745 über Medizinprodukte (Medical Device Regulation, MDR)

Software, die dazu bestimmt ist, Informationen zu liefern, die zu Entscheidungen für diagnostische oder therapeutische Zwecke herangezogen werden, gehört zur Klasse IIa, es sei denn, diese Entscheidungen haben Auswirkungen, die Folgendes verursachen können:

- den Tod oder eine irreversible Verschlechterung des Gesundheitszustands einer Person, in diesem Fall wird sie der Klasse III zugeordnet, oder
- eine schwerwiegende Verschlechterung des Gesundheitszustands einer Person oder einen chirurgischen Eingriff, in diesem Fall wird sie der Klasse IIb zugeordnet.

Software, die für die Kontrolle von physiologischen Prozessen bestimmt ist, gehört zur Klasse IIa, es sei denn, sie ist für die Kontrolle von vitalen physiologischen Parametern bestimmt, wobei die Art der Änderung dieser Parameter zu einer unmittelbaren Gefahr für den Patienten führen könnte; in diesem Fall wird sie der Klasse IIb zugeordnet.

Sämtliche andere Software wird der Klasse I zugeordnet.

## Footnotes

1 Recherche in PubMed am 30.01.2025 durchgeführt.

2 https://digital-strategy.ec.europa.eu/en/library/commission-publishes-guidelines-ai-system-definition-facilitate-first-ai-acts-rules-application, letzter Zugriff am 31.01.2025.

3 Produkt, dass zu einer in Anhang XVI MDR genannten Produktgruppe gehört.
